# Perception, Beliefs, and Attitudes Regarding Sedation Practices among Palliative Care Nurses and Physicians: A Qualitative Study

**DOI:** 10.1089/pmr.2021.0022

**Published:** 2021-05-24

**Authors:** Margaux Vieille, Lionel Dany, Pierre Le Coz, Sophie Avon, Charlotte Keraval, Sébastien Salas, Cécile Bernard

**Affiliations:** ^1^ Aix-Marseille Université, LPS, Aix-en-Provence, France.; ^2^ APHM, Timone, Service d'Oncologie Médicale, Marseille, France.; ^3^ Aix Marseille Université, CNRS, EFS, ADES, Marseille, France.; ^4^ Aix Marseille Université, CRO2, Marseille, France.; ^5^ Clinique Sainte Elisabeth, Marseille, France.

**Keywords:** interviews, palliative care, professionals, qualitative study, sedation

## Abstract

***Background:*** Palliative care teams face complex medical situations on a daily basis. These situations require joint reflection and decision making to propose appropriate patient care. Sometimes, sedation is one of the options to be considered. In addition to medical and technical criteria justifying the use of sedation, multiple psychosocial criteria impact the decision making of palliative care teams and guide, give sense to, and legitimize professional practices.

***Objective:*** The main goal of this study was to explore perceptions, experiences, and beliefs of palliative care teams about sedation practices in a legislative context (Claeys–Leonetti law, 2016; France), which authorizes continuous deep sedation (CDS) until death.

***Methods:*** This is a qualitative study using 28 semistructured interviews with physicians and nurses working in a palliative care team in France (PACA region). All verbal productions produced during interviews were fully transcribed and the contents analyzed.

***Findings:*** Content analysis revealed four themes: (1) sedation as a “good death,” (2) emotional experiences of sedations, (3) the practice of CDS, and (4) the ambiguous relationship with the Claeys–Leonetti law.

***Conclusions:*** This qualitative study provides evidence of a form of “naturalization” of the practice of sedation. However, the Claeys–Leonetti law exacerbates differences of opinion between palliative caregivers on sedation and questions the interest of this law for society and palliative care practices.

clinicalTrials.gov identifier: NCT04016038.

## Introduction

There is a consensus that physicians have an ethical obligation to relieve pain and other distressing symptoms in patients in an end-of-life condition.^1^ In the majority of cases, the severe symptoms can be controlled successfully. But in some cases, patients are faced with refractory symptoms: severe symptoms for which treatment is ineffective, or for which treatment will lead to unacceptable side effects.^2^ Sedation practices are used in some complex end-of-life situations and is one of the options when patients are faced with refractory suffering.^3^ Sedation in a palliative situation is defined as the attempt, by medicinal means, to induce a decrease in consciousness that may lead to a loss of consciousness. Its aim is to reduce or eliminate the perception of a situation experienced as unbearable by the patient, when all other means available and appropriate for this situation that could be proposed and/or implemented would not provide the expected relief. Sedation may be applied intermittently, transiently, or continuously.^4^

Sedation practices are the subject of extensive debate at international level.^5^ For example, there is a difference of perception regarding the hastening of a patient's death with continuous deep sedation (CDS).^6^^,^^7^ Some authors argue that it is morally wrong to decrease a patient's consciousness^8^ and that deep sedation causes the “social death” of the patient by altering his or her capacity to communicate. These debates illustrate the fact that despite extended theoretical or ethical discussion in the medical literature, the question of sedation practices remains a sensitive subject. Decision-making situations concerning sedation practices are often “decisive junctures”^9^ that bring together not only technical and medical but also psychological, social, relational, ethical, and even ideological criteria. Indeed, the patient's social context and personal preferences influence many professionals regarding sedation decisions and practices.^10–17^

Despite the important development of international studies in this field, we can observe a lack of research in France.^18^ Despite the long-standing development of palliative care in France, end-of-life conditions have been the subject of numerous ideological debates, political demands, and ethical and professional reflections. In 2005, the Leonetti law^19^ for end-of-life patients was adopted in France. It prohibits unreasonable obstinacy, obliges physicians to respect the patient's refusal to receive treatment, and asks that everything be done to relieve the patient's suffering (including sedation). In 2016, the Claeys–Leonetti law^20^ created new rights, in some cases granting terminally ill patients who refuse life-sustaining treatment the right to CDS until death. This new concept of CDS raises many questions especially at a time when public debate around the possibility of choosing conditions of death is ever present. This right to CDS was presented as a “French exception.”^21^ France is thus the first country to legislate on CDS and no study has been conducted so far concerning professional perception and experience in the context of the new Claeys–Leonetti law. Recognizing that sedation practice is situated in different legal contexts, the values and histories associated with palliative and end-of-life care may inform what are frequently contentious discussions about practice and policy on sedation.^8^ Moreover, sedation raises many questions for palliative care teams and involves emotionally engaging decision making and practice, regardless of the type of sedation practiced.^22–25^

The main purpose of this study was to clarify the role given to sedation practices in palliative care in France according to the new legal framework that authorizes CDS. This study was specifically aimed at:

Exploring the perceptions, experiences, and beliefs concerning the use of sedation practices and its contribution to the dying process.
Studying the process of accommodation that could occur for palliative caregivers confronted with new professional practices and their ethical issues.

## Methods

### Subjects

Our study involved caregivers working in palliative care teams in five hospitals in the Provence-Alpes-Côte d'Azur region (France). We included all genders, nurses, and physicians involved in sedation decision making or/and practices and agreeing to take part. Participants were selected at random from lists of the team's members provided by department heads. All caregivers were informed about the objectives and the design of the research and signed a consent form. They were asked to complete a sociodemographic questionnaire. Ethical approval for this research was obtained from the Ile de France VII Committee for the Protection of Persons (reference number: 2019-A00193-54).

### Method

Qualitative research involving semidirective interviews was carried out. This method helps to access the meaning given by the participants to the object studied. Interviews were led by health psychologists using a discussion guide containing focus guidelines and open questions (Table 1). The recorded interviews were transcribed verbatim. We conducted a content analysis of the data.^126^,^27^ The aim was to analyze the content of the interviews, divide it into units of meaning, and classify these units into categories according to analogical groupings. The units corresponded to themes and subthemes (Table 2). We obtained data saturation from the 16th interview. By means of interjudge evaluation, interviews were coded again with the thematic analysis structure stabilized. We found links between the different themes, through their subthemes, and we identified relationships between the different issues raised by the participants. The consolidated criteria for reporting qualitative research (COREQ) and checklist were used in the reporting of this study.^28^

**Table 1.** Interview Guide

Topics	Subtopics	Questions asked	Discussion aids
Sedation, emotions, and death	Perception about sedation Sedation and emotions Sedation in practice	What do you think about sedation? What is your experience of sedation situations? How do you approach sedation in your professional practice?	What does sedation mean to you? What do you think of sedation in palliative care? Of continuous deep sedation? What is a good death for you? What do you feel? What is your experience of the different sedation practices? Which sedation practices are easier to live with? What causes a different experience of sedation? What kind of sedation do you administer? How do you discuss sedation as a team? What is said to the patient? And how? How do you manage patient sedation requests?
The Claeys–Leonetti law	Law perception Law benefits and obstacles	What do you think about this law? What is the interest of this law for your practice?	What comparison do you make with the Leonetti law? How do patients and their families understand the law? How is it interesting? For patients? For relatives? For caregivers? On what points does it help you? How is it an obstacle to your practice? In what aspects?

**Table 2.** Summary of Thematic Analysis of Interviews

Themes	Subthemes explored	Keywords of interviews	Typical extracts
Sedation: the “good death”	(1) Question of falling asleep (2) Pain relief (3) Natural death with sedation (4) Therapy of last resort	Falling asleep with treatment Good conditions of death Naturally Last resort Pain relief	“Because we want to provide relief, but we know that something very serious is going on. So… it's dying in good conditions.” (physician, E14). “We stop artificial nutrition, well… we know that it will not continue for a long time… And it will remain a natural process […] there is no induction” (nurse, E5). “I'm convinced that sedation is really a form of care, a therapeutic option for exceptional situations. We're not all going to die with sedation… we don't need to sedate all our patients who are going to die” (physician, E10).
Sedation: emotions under stress	(1) Caregiver responsibility in sedation implementation (2) Temporality impact of dying process (3) Loss of communication and deep sedation (4) All sedation practices engage emotions	Significant Responsibility Slow/rapid death Complicated Interrupts interaction “Social death”	“Sedation is never simple. Because for me, it's something that matters, there's a heavy burden in those moments” (physician, E21). “I told myself that I was responsible for something and that the patient was not aware of the outcome […] it's still not without difficulties for nurses. It's us who make him/her unconscious at that time, not physicians” (nurse, E19). “It shouldn't last too long either… Once, I had a sedation that lasted three weeks. So it was complicated, for everyone, for everyone it was complicated” (physician, E11). “Deep and continuous sedation, which is considered by the law as a “social death.” It's social euthanasia, and in this, it interrupts any relationship between human beings” (physician, E22).
Understanding continuous deep sedation in practice	(1) Evaluate legitimacy of patient's request (2) Need for collective reflection (3) Ideal sedation: progressive and proportionate	Temporality Request analysis Care team discussion Repeated assessments Proportionate sedation	“I also believe that it's not because the patient says at the end of life “I'm tired of it, I want to die” … Also, you have to take the time to get the patient talking. There's this notion of temporality that's important” (physician, E7). “We still really discussed the legal conditions, that it was his choice, but that we also had to have some time to think about it, and in particular that it was a team decision and that we had meetings” (physician, E24). “And I told him “it's okay, we're not going to do things by halves. And so, we gave him a straight 5 mg […] But me, I like to give 2 mg, then wait a minute, then another 2 mg, then wait a minute… Even if it were an urgent sedation, even if the venous access had been in place, we could have done things more gently and more proportionately, administered a proportionate sedation” (physician, E12).
Ambiguous relationship to the Claeys–Leonetti law	(1) Barrier to euthanasia and sedative abuse (2) Complicates vs. oversees decision making and practices (3) Law responsible for societal confusions (4) A “hypocritical law”	Collegiality Protection Unnecessary law Hypocritical Vital prognosis Criteria	“We are in a service where the law protects us, the law protects our decisions, it oversees our decision-making, because we need it. Because families and patients' requests are clear, unclear… we make projections despite ourselves…” (nurse, E28). “This new law is causing trouble. It has been publicized. The problem with the patient is ‘I have the right to deep and continuous sedation!’ Because it was said a bit like that, but without knowing the criteria for being entitled to it either” (nurse, E16). “I think it's a bit vague to specifically impose reflection on us, to impose collegiality on us. Because if it's not ambiguous, I don't need collegiality.” (nurse, E2). “I find that as the law proposes deep and continuous sedation until death, but only using the sedation guidelines, this is a false promise. We induce sleep and a few hours later… they wake up. And that wasn't what we agreed with the patient or with his/her family” (physician, E17).

## Findings

A total of 28 caregivers (14 physicians and 14 nurses) agreed to participate and gave informed consent. In our sample, we observed a large proportion of women (*n* = 25). The average age of participants was 40.9 years [standard deviation (SD = 8.40)], ranging from 26 to 60 years. The average length of service was 12.6 years (SD = 8.73), ranging from 1 to 32 years, and the average length of service in palliative care was 6.52 years (SD = 5.05), ranging from 1 month to 20 years (Table 3).

**Table 3.** Sociodemographic Characteristics of the Population (*N* = 28)

	Physicians (*n* = 14)	Nurses (*n* = 14)	Total (*n* = 28)
Characteristic	Frequency (%)		
Gender			
Female	13 (46.4)	12 (42.85)	25 (89.3)
Male	1 (3.57)	2 (7.14)	3 (10.7)
Age (years)			
<30	1 (3.57)	1 (3.57)	2 (7.14)
30–49	10 (35.7)	11 (39.28)	21 (75)
>50	3 (10.7)	2 (7.14)	5 (17.85)
Length of service (years)			
<10	7 (25)	4 (14.28)	11 (39.28)
10–25	5 (17.85)	8 (28.6)	13 (46.4)
>25	2 (7.14)	2 (7.14)	4 (14.28)
Length of service in palliative care (years)			
<1	1 (3.57)	2 (7.14)	3 (10.7)
1–5	4 (14.28)	5 (17.85)	9 (32.14)
6–10	6 (21.4)	6 (21.4)	12 (42.85)
>10	3 (10.7)	1 (3.57)	4 (14.28)

### Sedation: The “good death”

Professionals seem to perceive sedation as a way of allowing patients to die in good conditions. Some participants (11/28) describe sedation as a therapy that reduces the patient's state of consciousness by medicinal means, either temporarily or deeply. By inducing this temporary or deep sleep, they (17/28) explain that they seek to reduce or relieve patients' suffering (“personally, I think it's an alternative that provides relief for patients” [physician, E25]). Some professionals associate words such as “dying,” “going away,” or “leaving” with the existence of the risks of dying while falling asleep (“it's certain that… it's leaving quietly, without pain” [physician, E24]). But professionals specify that it is not an induced and not an intentional death. For these professionals (10/28), it is important to associate sedation with a “natural death” (“Sedation doesn't kill the patient. It's a natural death. He/she is going to die a natural death, that's the big difference!” [nurse, E27]). Finally, for some participants, it is important to describe sedation as a therapy of last resort (8/28), when everything else has been tried (“Sedation is exceptional! I always start my class by saying ‘sedation is an exceptional practice,’ because that's the reality” [physician, E21]).

### Sedation: Emotions under stress

Although sedation is perceived as a “good way” to die, it does not necessarily mean that it is a good emotional experience for caregivers. Sedation practice generates a sense of responsibility for caregivers (10/28), with sometimes a feeling of hastening the death of the patient, like euthanasia (“Sometimes you feel like you're killing someone, because deep inside us we're thinking, and we know it's for their own well-being… But it's still you, at this moment, injecting a syringe of Hypnovel…” [physician, E3]). The temporality of the patient's end of life can affect the caregiver's experience (10/28). A quick or slow death leads to questions and uneasiness among caregivers. A quick death after sedation induction may shorten contact with relatives or prevent the introduction of a collective reflection. However, an extended sedation is also difficult, because death becomes difficult to bear for caregivers and patients' relatives (“I said to myself ‘God… it's hard to wait for the death of your loved one. To wait a day, two days…ok. But waiting a week, two weeks […] I found it very painful and very difficult’” [nurse, E28]). Loss of communication in deep sedation is another element that causes caregivers to question the sense of patient's end-of-life conditions (9/28). Indeed, for caregivers, relationships with patients are an essential ingredient of quality palliative care. In the end, a majority of professionals (15/28) explain that all sedative practices engage their emotions.

### Understanding CDS in practice

A key feature of the Claeys–Leonetti law is that patients have the right to request a CDS from the palliative care team. Half of the caregivers express the need to discuss as a team each patient's request (regardless of the nature of this request), and wonder whether this request is justified. This reflection can lead to the development of alternative reasons (pain, isolation) at the request of the patient. Ten caregivers report the necessity to make time for a collective reflection even if it means not responding immediately to the request (“We have the right to say to the patient ‘I have heard about your suffering or your request but the team has to take time to see if we can access this.’ And here, this introduces another issue of temporality into sedation, which is collegial reflection” [physician, E25]). Finally, in practice, regardless of the type of sedation, caregivers explain that they prefer to administrate proportionate and progressive sedation (9/28) (“I know that for me it's complicated to administer deep sedation… it's true that I tend to sedate rather progressively” [physician, E24]). These sedation practices seem to have to comply with some professional norms to be perceived as good practice. Progressive sedation seems to be an ideal sedation. However, this sedation is not a new practice for these professionals, and it has already complied with the conditions for a “good death.”

### Ambiguous relationship with the Claeys–Leonetti law

When professionals talk about this law and especially about the French exception concerning CDS, their opinions differ. For 9 of 28 caregivers, this law has helped to avoid the legalization of euthanasia in France, while overseeing some sedation practices to avoid some abuses, such as passive euthanasia. (“I think this law is trying to save time before a pro-euthanasia law” [physician, E17]). Some professionals (18/28) explain that this law oversees or complicates sedation decision making and practice. This law provides protection for caregivers, requires teams to think about some situations, and guides their practices (“It's even good that it's overseen from a legislative point of view because it has enabled us to work with more peace of mind” [physician, E4]). However, for some professionals, this law complicates practices. Five caregivers express the opinion that the previous law^19^ was sufficient (“We didn't need that. The 2005 law was good enough” [physician, E15]). For others, criteria of short-term vital prognosis are difficult to evaluate, a collegial procedure can be difficult to implement in some situations and this law does not answer real patients' requests (“We'll explain the law, but in other words, so, we'll change the patient's mind. Sometimes, we are spectators of this” [nurse, E19]). In addition, this law is described as “hypocritical” (7/28) for several reasons: the lack of conviction, the patient's social death, not being the answer to a request to die, and the attitude of ignoring the impact of some medications. Finally, this law creates societal confusions over sedation and euthanasia (14/28) (“We had a patient who came with his family, and who told us “the law allows me to be sedated, I want to be sedated. We realized that the patient had not understood the law” [nurse, E8]).

## Discussion

Our findings show that participants have a common representation about sedation, even though different terms and practices exist. Sedation is described as a therapy that has necessary criteria to guarantee patients a good end of life, falling asleep, pain relief, and a natural death. This notion of “peaceful sedation” has been found in other studies that have investigated English and Belgian caregivers' perceptions of sedation.^12,13^ The criteria of a “natural” death helps to differentiate sedation from other practices (euthanasia; assisted suicide). As Seymour et al. wrote in a study about Dutch caregivers,^8^ for our caregivers, sedation does not hasten the patient's end of life, the patient's death is the result of the evolution of the disease. Nevertheless, for others, sedation is an alternative to euthanasia that facilitates a natural and peaceful death.^13^ Therefore, caregivers' perceptions about the relationship between sedation and patient death are different regardless of the country and its legal context. However, French caregivers emphasize the difference between sedation and other practices, and especially the “natural” aspect of death and the noninduction of death by sedation. In addition, professionals insist on the fact that sedation is a therapy of last resort. In a Swiss study,^22^ palliative care professionals also highlighted the exceptional nature of this therapy. This element illustrates the professional's ambiguity about the responsibility of using sedation in the patient's death: if sedation guarantees a natural process and does not kill the patient, why do they need to underline the exceptional aspect of this practice? These findings show that caregivers need to think of the end-of-life process as natural to legitimize their professional practices.

Even if the caregiver's intention is not to hasten the death of the patient, involvement in this act is not without emotional consequences, and creates a sense of responsibility among caregivers in the patient's end of life.^14^ This perceived responsibility is even greater when the time between the implementation of sedation and the patient's death is short.^23^ In addition, emergency sedation raises many moral concerns among caregivers who feel they are administering it without a time for collective reflection.^22^

In a way, we are witnessing the expression of a norm concerning the “right temporality” for dying during sedation. In France, the Claeys–Leonetti law proposes CDS as a new therapeutic option. For caregivers, CDS creates a patient's “social death,” making it impossible to communicate with others. This new situation questions the sense of end-of-life conditions and palliative practices. For caregivers, it is important to maintain interaction between patients and relatives because communication is an essential element in palliative care.^24,25^ Therefore, to maintain contact, caregivers explain that they prefer progressive sedation to CDS. Leboul^14^ developed this idea: loss of communication is experienced for caregivers as a failure, compared with an ideal situation wherein the patient would die while maintaining contact until the end of life.

As we highlighted in this study, Ziegler et al.^22^ also explained that it is necessary to make time for discussion between professionals to reappraise this request. Some caregivers perceived that a request for CDS could cause them psychological discomfort.^14^ Therefore, in our study, caregivers explained that they impose a time for reflection on patients to validate their decisions. Finally, despite the existence of a law proposing CDS, they explained that they prefer to implement progressive sedation. These findings are close to others (in countries with a different legislative context) that observed that caregivers explain that they are more comfortable with progressive sedation than CDS.^10,11,22–29^ More specifically, for Swart et al.,^24^ deep sedation can be gradual and progressive, and they report perceived death as more natural with progressive sedation. To conclude, progressive and proportionate sedation is enough and perceived as the “ideal” sedation. Progressive sedation meets the characteristics of a “good death” by sedation.

The Claeys–Leonetti law is specific to the French legislative context, and this country is the only one to have legislated for CDS.^21^ A majority of French palliative care professionals are opposed to the legalization of euthanasia.^15^ CDS is an alternative because the French population asked for new rights for patients' end of life. In that sense, for our caregivers, this law has helped to regulate professional practices by limiting the improper use of sedation and preventing a law on euthanasia. In addition, it helps to protect health care teams in relation to certain decisions/actions and imposes a time for ethical reflection. Conversely, for others, this law has complicated their professional practices and their relations with patients and relatives. These caregivers explain that it is impossible to follow guidelines, especially in an emergency situation. For them, criteria such as a “short-term vital prognosis” for implementing sedation are difficult to evaluate. In addition, it is not always possible for caregivers to guarantee that patients will not wake up during sedation. This element emerges in a study^24^ that demonstrates the discomfort of Dutch caregivers toward these awakenings. Our findings show, as do those of Robijn et al.,^10^ that patients and families often have a lack of knowledge about sedation legislation causing mistaken beliefs. Finally, regardless of country and legislation, sedation creates normative tensions and differences of opinion. Even though, for a long time, sedation has legitimized the practices and the expertise of palliative care professionals,^16^ this new French legalization weakens the common identity that the community of palliative care professionals are striving to maintain.^29^

These findings demonstrate the value of establishing working groups for palliative care teams on emotional management, so that they can experience these practices better. But also, ethics training on sedation practices could be proposed, so that caregivers can reflect together on the basis of lived practices. There were limitations to our study. Participants were selected from one geographic region, and their experiences and opinions may not be generally applicable. Furthermore, we only interviewed physicians and nurses without considering the point of view of other caregivers and our sample interviewed was composed mainly of women.

## Conclusion

This is the first qualitative interview-based research carried out in France, exploring the perceptions, experience, and beliefs of palliative care professionals about sedation practices, particularly about CDS. This analysis shows that in practice, progressive and proportionate sedation remains the most often administered, despite the existence of CDS, because, in the opinion of caregivers, this type of sedation fulfills the criteria of a “good death” with sedation. The recent Claeys–Leonetti law exacerbates differences of opinion between palliative caregivers about sedation and questions the interest of this law for society and palliative care practices. This study has proven the existence of a subgroup in the French palliative care community, in which a collective identity and ideology are highlighted.

## Abbreviations Used

CDS: continuous deep sedation
COREQ: consolidated criteria for reporting qualitative research
PACA: Provence-Alpes-Côte d'Azur
SD: standard deviation

## References

[1] Cherny NI, Portenoy RK: Sedation in the management of refractory symptoms: Guidelines for evaluation and treatment. J Palliat Care 1994;10:31–38 8089815

[2] van Dooren S, vanVeluw HT, van Zuylen L, et al.: Exploration of concerns of relatives during continuous palliative sedation of their family members with cancer. J Pain Symptom Manage 2009;38:452–459 19559563 10.1016/j.jpainsymman.2008.11.011

[3] Quill TE, Byock IR: Responding to intractable terminal suffering: The role of terminal sedation and voluntary refusal of food and fluids. Ann Intern Med 2000;132:408–414 10691593 10.7326/0003-4819-132-5-200003070-00012

[4] Sedation for terminal distress and in specific and complex situations: Recommendations in adults and specificities at home and in geriatrics. Société française d'accompagnement et de soins palliatifs (SFAP). 2014. http://www.sfap.org/system/files/sedation-phase-terminale.pdf (Last accessed 95, 2020)

[5] Booker R, Bruce A: Palliative sedation and medical assistance in dying: Distinctly different or simply semantics? Nurs Inq 2020;27:e12321 31756038 10.1111/nin.12321 PMC9285680

[6] Maeda I, Morita T, Yamaguchi T, et al.: Effect of continuous deep sedation on survival in patients with advanced cancer (J-Proval): A propensity score-weighted analysis of a prospective cohort study. Lancet Oncol 2016;17:115–122 26610854 10.1016/S1470-2045(15)00401-5

[7] Raus K, Chambaere K, Sterckx S: Controversies surrounding continuous deep sedation at the end of life: The parliamentary and societal debates in France. BMC Med Ethics 2016;17:36 27357285 10.1186/s12910-016-0116-2 PMC4928322

[8] Seymour J, Rietjens J, Bruinsma S, et al.: UNBIASED consortium: Using continuous sedation until death for cancer patients: A qualitative interview study of physicians' and nurses' practice in three European countries. Palliat Med 2015;29:48–59 25062816 10.1177/0269216314543319 PMC4266692

[9] Glaser BG, Strauss AL: Time for Dying. Chicago: Aldine, 1980

[10] Robijn L, Chambaere K, Raus K, et al.: Reasons for continuous sedation until death in cancer patients: A qualitative interview study. Eur J Cancer Care 2017;26:e12405 10.1111/ecc.12405 26515814

[11] Blondeau D, Dumont S, Roy L, Martineau I: Attitudes of Quebec doctors toward sedation at the end of life: An exploratory study. Palliat Support Care 2009;7:331–337 19788775 10.1017/S1478951509990265

[12] De Vries K, Plaskota M: Ethical dilemmas faced by hospice nurses when administering palliative sedation to patients with terminal cancer. Palliat Support Care 2017;15:148–157 27323872 10.1017/S1478951516000419

[13] Sercu M, Pype P, Christiaens T, et al.: Belgian general practitioners' perspectives on the use of palliative sedation in end-of-life home care: A qualitative study. J Pain Symptom Manage 2014;47:1054–1063 24095283 10.1016/j.jpainsymman.2013.06.016

[14] Leboul D: Sedation in palliative care. Opportunity to think about the ethics of a clinic of uncertainty? In: Jacquemin D (ed): Sedation, Euthanasia: Ethics and Spirituality for Thinking. Namur, Paris: Lumen vitae, Collection Trajectoires n°30, éditions jésuites, 2017, pp. 199–217

[15] Dany L, Baumstarck K, Dudoit E, et al.: Determinants of favourable opinions about euthanasia in a sample of French physicians. BMC Palliat Care 2015;14:59 26542685 10.1186/s12904-015-0055-6 PMC4635994

[16] Peretti-Watel P, Bendiane MK, Moatti JP: Attitudes toward palliative care, conceptions of euthanasia and opinions about its legalization among French physicians. Soc Sci Med 2005;60:1781–1793 15686809 10.1016/j.socscimed.2004.08.037

[17] Swart SJ, van der Heide A, van Zuylen L, et al.: Continuous palliative sedation: Not only a response to physical suffering. J Palliat Med 2014;17:27–36 24410419 10.1089/jpm.2013.0121

[18] Claessens P, Menten J, Schotsmans P, Broeckaert B: Palliative sedation: A review of the research literature. J Pain Symptom Manage 2008;36:310–333 18657380 10.1016/j.jpainsymman.2007.10.004

[19] Loi n° 2005-370 du 22 avril 2005 relative aux droits des malades et à la fin de vie. www.legifrance.gouv.fr (Last accessed 415, 2019)

[20] Loi n° 2016-87 du 2 février 2016 créant de nouveaux droits en faveur des malades et des personnes en fin de vie. www.legifrance.gouv.fr (Last accessed 415, 2019) 30512616

[21] Horn R: The “French exception”: The right to continuous deep sedation at the end of life. J Med Ethics 2018;44:204–205 29056584 10.1136/medethics-2017-104484 PMC5869460

[22] Ziegler S, Merker H, Schmid M, Puhan MA: The impact of the inpatient practice of continuous deep sedation until death on health care professionals' emotional well-being: A systematic review. BMC Palliat Care 2017;16:30 28482856 10.1186/s12904-017-0205-0 PMC5422916

[23] Raus K, Brown J, Seale C, et al.: Continuous sedation until death: The everyday moral reasoning of physicians, nurses and family caregivers in the UK, The Netherlands and Belgium. BMC Med Ethics 2014;15:14 24555871 10.1186/1472-6939-15-14 PMC3942295

[24] Swart SJ, van der Heide A, van Zuylen L, et al.: Considerations of physicians about the depth of palliative sedation at the end of life. CMAJ 2012;184:E360–E366 22331961 10.1503/cmaj.110847 PMC3328540

[25] Abarshi EA, Papavasiliou ES, Preston N, et al.: The complexity of nurses' attitudes and practice of sedation at the end of life: A systematic literature review. J Pain Symptom Manage 2014;47:915–925.e11 10.1016/j.jpainsymman.2013.06.011 24075400

[26] Bauer MW: Classical content analysis: A review. In: Qualitative Researching with Text, Image and Sound: A pratical Handbook. London: SAGE, 2000, pp. 131–151.

[27] Flick U: Thematic coding and content analysis. In: An Introduction to Qualiative Research. London: SAGE, 2014, pp. 420–438.

[28] Tong A, Sainsbury P, Craig J: Consolidated criteria for reporting qualitative research (COREQ): A 32-item checklist for interviews and focus groups. Int J Qual Health Care 2007;19:349–357 17872937 10.1093/intqhc/mzm042

[29] Castra M: Dying Well: Sociology of Palliative Care. Paris: PUF, 2003

